# Cell Cytometry: Review and Perspective on Biotechnological Advances

**DOI:** 10.3389/fbioe.2019.00147

**Published:** 2019-06-18

**Authors:** Abhishek Vembadi, Anoop Menachery, Mohammad A. Qasaimeh

**Affiliations:** ^1^Division of Engineering, New York University, Abu Dhabi, United Arab Emirates; ^2^Department of Mechanical and Aerospace Engineering, New York University Tandon School of Engineering, Brooklyn, NY, United States

**Keywords:** cytometry, enumeration, biotechnology, microfluidics, microfabrication

## Abstract

Cell identification and enumeration are essential procedures within clinical and research laboratories. For over 150 years, quantitative investigation of body fluids such as counts of various blood cells has been an important tool for diagnostic analysis. With the current evolution of point-of-care diagnostics and precision medicine, cheap and precise cell counting technologies are in demand. This article reviews the timeline and recent notable advancements in cell counting that have occurred as a result of improvements in sensing including optical and electrical technology, enhancements in image processing capabilities, and contributions of micro and nanotechnologies. Cell enumeration methods have evolved from the use of manual counting using a hemocytometer to automated cell counters capable of providing reliable counts with high precision and throughput. These developments have been enabled by the use of precision engineering, micro and nanotechnology approaches, automation and multivariate data analysis. Commercially available automated cell counters can be broadly classified into three categories based on the principle of detection namely, electrical impedance, optical analysis and image analysis. These technologies have many common scientific uses, such as hematological analysis, urine analysis and bacterial enumeration. In addition to commercially available technologies, future technological trends using lab-on-a-chip devices have been discussed in detail. Lab-on-a-chip platforms utilize the existing three detection technologies with innovative design changes utilizing advanced nano/microfabrication to produce customized devices suited to specific applications.

## Introduction

Precise determination of cells in a sample is important for a broad field of applications, such as tissue culture studies by microbiologists and disease progression in medical laboratories (OYAMA and EAGLE, 1956; Houwen, 2001). Early medical practitioners realized the importance of counting blood cells as a tool for investigation and quantitative study in healthcare (Verso, 1964). Prior to the advent of microscopy, bacterial microorganisms were enumerated with the naked eye by means of colony counts. This is restricted by culture growth time since most microorganisms take long to form visible colonies. Subsequently, cells were counted manually using a microscope. For over 100 years, cell biologists have been using a hemocytometer (counting chambers) to quantify cells (Bard, 1974). This lets the user count the number of cells manually, within a volume of liquid, under a microscope. Mass manufacturing techniques led to addition of laser etched Neubauer grids in modern slides and made them cheaper (available retail for < $ 100 from Sigma Aldrich). Despite the gradual replacement of manual counting methods with automated counters due to their ability to process multiple specimen types including blood and urine, hemocytometer is still a mainstay of all cell biology laboratories as of now (Hsiung et al., 2013).

Nowadays, there are three main commercial methods (detection principles) used in automated counters. The first is the automated detection and analysis using electrical properties as the basis of distinguishing cells. Coulter counter uses the impedance properties of the cell to make the analysis; cells are detected every time they cross through an aperture (orifice), typically around a few micrometers in size. Modern coulter counters are capable of measuring cells even smaller than 1 μm (Yang and Yamamoto, 2016). Bacterial cells, typically much smaller than mammalian cells, can be detected with coulter counters. Coulter counter was the first electronic cell counter which was widely accepted as an alternative to manual counting. Bull et al. showed clinical relevance of early coulter counters in making accurate and reproducible platelet counts (Bull et al., 1965).

Optical Flow cytometer, another widely used commercial method available in the market, is based on optical cell detection (Lee et al., 2003). It is used for management of patient diagnosis and prognosis, such as for blood cancers like leukemia and Lymphoma (Jelinek et al., 2017). They provide large scale, automated quantification of cells based on their optical parameters on a cell by cell basis. Laser light is illuminated on the particles in the sample stream; thereby, getting scattered and measured by optical detectors. Each event or cell in the flow produces characteristic information or parameter based on its light scattering and/or fluorescent properties, hence optical flow cytometers can simultaneously provide multi-parametric analysis with data on cell morphology and chemical characteristics (Cho et al., 2010). Several optical flow cytometers have an added feature which allows them to physically separate cells of interest from a heterogeneous sample, based on their optical properties and are categorized as Fluorescence assisted cell sorting (FACS) systems. They have a low coefficient of variation (CV) when counting cells below the range of 30 cells/μL (Sandhaus et al., 2010). Krediet et al. reported high precision and accuracy while using optical flow cytometer in comparison to manual and coulter counting methods (Krediet et al., 2015). They used Millipore Guava™ flow cytometer for counting algal cells and the device was able to perform quantification over a wide range of cell concentrations. The system provides automated processing (rinsing and mixing) of 96-well plate samples providing low operator time and high throughput. The Food and Drug Administration (FDA) has approved Flow cytometry as a clinical detection technique which will help manufacturers target more devices for clinical hematology and circulating tumor cell (CTC) detection (Goodin, 2017). Flow cytometers and Coulter counters have earned a place in research and clinical laboratories despite their bulky size and costs, which is explained due to the rise in demand for precision high-end cell counters for high throughput counts. In general, these devices can cost up to tens of thousands of dollars, which makes them unfeasible for developing countries or for portable point-of-care diagnostics.

The third most common type of device utilizes digital image analysis using a photo-microscope (O'Brien et al., 2016). These devices, readily available from established manufacturers like ThermoFischer scientific (i.e., Countess II automated cell counter) and BioRad (TC20 automated cell counter), contain an optical system with autofocus, capable of detecting cells using integrated software to characterize particles in terms of their fluorescent intensity and size while having a processing time of few seconds (Goldberg, 2008; Gupta et al., 2018). Image cytometry devices can either analyze static samples or perform kinetic analysis, in which case it is also referred to as imaging flow cytometry (Lanigan et al., 1993). Imaging flow cytometry gained commercial use after technological improvements in optics and detection systems that allowed the imaging of moving cells to stay in focus. There are some examples of small full blood counting systems utilizing image cytometry, such as HemoCUE WBC system, which can make cell counts with clinical accuracy and have been certified by the European Conformity CE marking (Lohman et al., 2018). Lui et al. developed a method for CTC detection in hepatocellular carcinoma using image flow cytometry (Liu et al., 2016). They performed fluorescent imaging of cells and enumeration of CTCs based on morphological criteria. In research laboratories, images of cells obtained using microscopy are also commonly analyzed and quantified using open source software (Hennig et al., 2017). Cell profiler, ImageJ, and other such open-source software, are routinely used by cell biologists for cell count from thousands of images, by performing object identification through segmentation, thresholding, recognition and division of clumped cells and other processing steps, which are user customizable.

A brief comparison of the different counting methods is summarized in Table 1. Values of sample concentration for various devices obtained by correspondence from company.

**Table 1:** Summary of commercial cell counting principles and specifications.

**Detection principle**	**Example**	**Sample concentration (cells/mL)**	**Price range ($)**	**Label needed**	**Analysis time/throughput**
Manual counting	Sigma-Aldrich, BrightLine Hemocytometer	2 × 10^5^-2.5 × 10^6^	$	Yes—Trypan Blue, Methylene Blue, Erythrosin B, Nigrosin, Saffrarin	Low
	SKC,Inc, C-Chip™ Disposable Hemacytometers				
Impedance system	Scepter 2.0- Sigma-Aldrich	2 × 10^3^-1 × 10^6^	$$-$$$	NO	High
	Multisizer 4 COULTER COUNTER				
Optical system	ACCURI c6- BD	1 × 10^3^-1 × 10^6^	$$$	Both- Label free for scattering ; Label Needed for absorption and fluorescence	High
	Guava- Millipore				
Image cytometer	Countess II-Fischer scientific	1 × 10^4^-1 × 10^7^	$$	Yes- for cell identification	Low
	Cellometer T4- Nexcelom				

**Figure 1:**
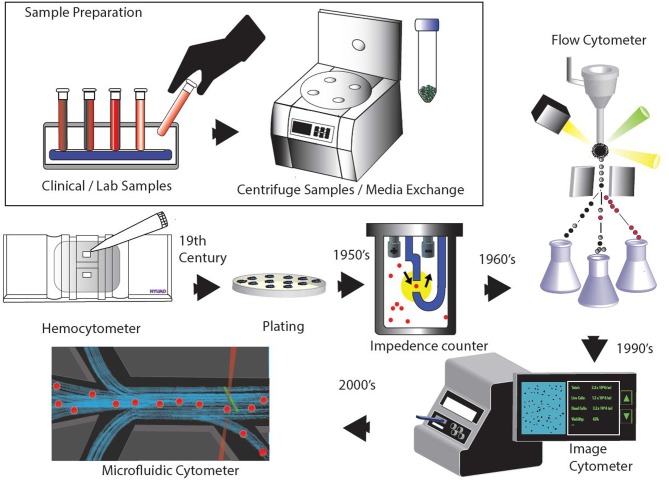
Evolution of various cell counting and detection technologies.

With the rise of microfluidic devices, several attempts have been made to develop cell counters embedded in a chip. These devices show great promise as portable and low-cost diagnostic tools (Chin et al., 2012). Microfluidics finds itself organically linked with cell manipulation and analysis because of the obvious size match between microfluidics- leveraging micrometer scale channels to process low volume fluid samples (Whitesides, 2006). However, improved stability over longer periods of time is necessary for the technology to become mainstream and find commercial success. Many studies have been done for sample enumeration in a single microfluidic platform (van Berkel et al., 2011). Currently, most of the microfluidics based cell counting systems available in the market, such as FX500 by Sony Biotech and Ampha z32 by Amphasys, use a microfluidic chip or cartridge integrated within conventional cytometry system. Such designs offer simplicity and flexibility since they feature a replaceable fluidics system with microchannels for sample flow, which is easy to set-up and maintain. For microfluidic devices to supplant current cytometers, it will require more development in sample preparation and detection techniques within a lab-on-a-chip microfluidic platform. The timeline in Figure 1 shows an overview of all cell counting methodologies.

## Clinical Significance

Clinics and hospitals worldwide widely use cell counting to determine the health of a patient. Hematological analysis of body fluids can provide valuable diagnostic information and indicate medical conditions. Physician can use the results from these tests to learn a great deal about health and help in discovering the problem in its early phase. Since blood is rich with cells and proteins, among other biological substances, and provides important information about health, it has become a form of liquid biopsy performed on patients for cases such as monitoring an anemia, infection, cancer and a wide gamut of disorders. The full blood count is therefore routinely performed for any measure of diagnoses in clinics and it involves counting all the cells in the blood sample (Houwen, 2001). This determines the composition of various constituents within the blood such as red blood cell (RBC), white blood cell (WBC), and platelets, which give insight into the health status of a patient. For example, low white blood cell count could be a cause of a bigger underlying issue like infections with Human immunodeficiency virus (HIV), which uses CD4+ T Lymphocyte cells for replication and hence leads to critically low WBC count (Kannel et al., 1992). Hence, methods for CD4+ T cell enumeration are important for HIV disease staging and treatment monitoring, which makes portable label-free cell cytometers a necessity especially in resource limited setting where the disease has a higher prevalence (Cheng et al., 2007). Multi-parametric flow cytometry has become the mainstay of most hematologic malignancies (Braylan, 2004) and automated counters are standard inventory of most medical diagnostic laboratory. Automated cell counters like Sysmex XE-200 or Beckman coulter DxH 600 are extensively used by laboratory technologists for analyzing most body fluid samples due to their low turnaround times and improved precision, as opposed to manual hemocytometer. Other instruments such as the CSF cell counter GlowCyte by Advanced Instruments are marketed for specialized sample analysis in clinics and offer comprehensive results with patient reports. These devices follow validation and verification protocols set by international regulations, like the FDA in USA and diagnostic devices directive in EU, to ensure universal standards are set for the laboratory method (Verbrugge and Huisman, 2015). Cell counting and analysis of CTCs is also a widely popular field of research due to the prognostic information in cancer patients. For example, it was shown that the detection of CTCs using flow cytometry can be a predictor of survival in patients (Nowakowski et al., 2005). The use of FDA approved CTC enumeration by CellSearch™ (Menarini Silicon Biosystems) opened the door for rare cell characterization for drug development and as a prognostic marker for personalized therapies which has led to several such attempts at quantification of rare cells (Maertens et al., 2017).

## Hemocytometer

After the modern hemocytometer was initially invented in the late nineteenth century, it has been used extensively for cell enumeration. The device evolved from being used by clinical practitioners to analyze blood samples to becoming a common tool in laboratories and clinics all over the world. It is still commonly used for counting cells due to its low cost and versatility.

The history of hemocytometer is intertwined with hematology, as the study of blood associated diseases was of interest to the early physicians (Verso, 1964). The first such device which resembled the modern hemocytometer was invented by Louis Charles Malassez in 1874 and this was followed by several iterations to make improvements to the design. He created a counting chamber glued to glass slide with markings that correlated the length to volume of sample. The concentration of cells could be determined by counting them and multiplying by the dilution factor. The most significant improvement to the counting efficacy was proposed by Karl Burker (1872-1957), who made the device using heavy glass slide and three cemented glass platforms on the surface along with gridding. Over time, the hemocytometer rulings and counting grid evolved to account for more diverse requirements; initial design was made for counting smaller cells, like RBCs, and was not appropriate for other kinds of cells.

**Figure 2:**
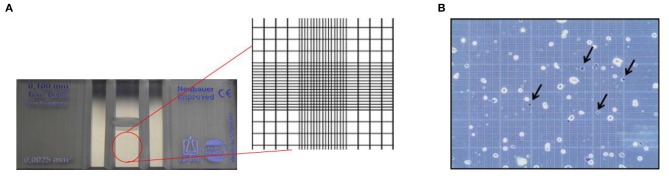
Principle of hemocytometer for cell counting. **(A)** Zoomed inset of a hemocytometer showing the Neubauer chamber with counting grid. **(B)** Stained colon carcinoma CT-26 cells using trypan blue dye where the arrows indicate dead cells (Hong et al., 2011). This work is licensed under the Creative Commons Attribution 4.0 International License.

Hemocytometers currently available in the market are mass manufactured with laser etched grids. They are more accurate and easier to use compared to its predecessors. The device is crafted to ensure that the area covered by the lines as well as the volume of the chamber is known. The gridded area of the hemocytometer consists of 1×1 mm squares, which are again subdivided into smaller divisions, as shown in Figure 2A. The cell suspension is loaded via capillary action into the chamber and the cells are counted manually. The number of cells can further be used to determine the concentration or density of the cells (Absher, 1973).

Traditionally, a hemocytometer was also used to supplement cell enumeration with viability analysis, shown in Figure 2B. Cell viability, which is essential to assess the kinetics of growth, is performed by the selective staining of the sample using various staining approaches. Vital exclusion stains are commonly used to mark cells that have lost membrane integrity such as Trypan Blue or inclusion stains which mark cells retaining membrane integrity such as Fluorescein Diacetate (Kwizera et al., 2017). Hemocytometer is not limited to certain staining methods and allows all staining techniques according to the purpose of the analyses. Variations of the device, such as Nageotte hemocytometer allow analysis of low cell concentration beyond the capabilities of standard hemocytometer. Nageotte hemocytometer have been shown to make accurate counts of low leukocyte concentrations using crystal violet stain (Lutz and Dzik, 1993).

Although an integral part of every laboratory in cell based research, the method is still subject to several inherent shortcomings due to its design. Instrumentation and material variation, such as the type of buffer and pipette used, can add unwanted errors to the result. Since hemocytometer still depends on manual counting, human error is inevitable in the various stages of the process (mixing, handling, and dilution). If the analyst does not have a certain level of expertise in using the hemocytometer, it may lead to less accuracy and high inter-operator errors. The standard error in a hemocytometer is described as $1/\sqrt{}n$, where *n* is the number of cells counted (Berkson et al., 1939). Biggs et al. performed a study to quantify the standard and observer error using trained doctors who were made to count RBCs. Operator and standard error was reported to be 3.12 and 7.8%, respectively (Biggs and Macmillan, 1948). The cells have to be diluted and mixed thoroughly, to ensure that the sample is representative of the original mixture and to ensure it is not too crowded and difficult to count. Higher dilution factors also seemed to produce higher standard error in the results obtained (Ongena et al., 2010). Multiple sample counts usually need to be made to ensure the results are consistent and representative of the original sample. The manual counting process is also time consuming and hence not efficient to be implemented for large scale analyses. Despite the shortcomings, in the hands of an experienced user it continues to be a useful tool to discriminate and enumerate a heterogeneous population of cells.

## Automatic Cell Counting

Since the early days of cell counting, there were attempts made to automate the process to yield high throughput, while maintaining accuracy. With the development of computers, improved optical and electronic components, cameras and algorithms, it is possible to eliminate some of the errors of manual counting through automation in the cell counting process. Fully automated systems can also perform sample preparation thereby reducing operation and turnaround time.

### Electrical Impedance Based Cell Counting

Cell analysis using impedance for counting particles is one of the most popular conventional methods. It is predominantly based on the “Coulter principle” which was developed by Wallace H. Coulter, in 1953, who reported a novel method to analyze individual cells in a fluidic channel (Simson, 2013). After his invention of impedance based single cell flow cytometer, he focused its usage on counting RBCs since he believed automation was desirable in clinics due to error prone measurements in manual cell counting and user dependent variability. Wallace described it as a non-optical counting system, and claimed it could count over 6,000 single cells per second. It was a revolutionary device at that time, since most diagnostic laboratories had physicians working tediously on microscopes to count blood cells on hemocytometers. His invention was pitched as an alternative to visual cell counting and quickly commercialized as an instrument (Coulter, 1956).

The Coulter principle is based on the phenomenon that the electrical impedance of a particle is different from that of the buffer. An aperture is placed between two electrodes while the electrolyte forms the current path. The aperture creates a “sensing zone,” which causes a change in impedance, concurrent with electric current, as the particle passes through it (Kubitschek, 1969). Furthermore, the change in impedance is correlated to the size of the particle and its volume which is the foundation of cell size analysis (Hurley, 1970). The change of impedance within an aperture depends on various physical aspects such as particle size, shape and orientation. In addition to visible physical attributes of the particle, the electrical conductivity of the particle and solution, and homogeneity of the electric field also have a significant effect on impedance. Since the movement of particles across this aperture causes a decrease in the electric current passing through, these pulses can be monitored to count the particles. Also, because this depends on the intrinsic electrical properties of the cell, a fluorescent marker or label is not necessary. The initial design of the device, as illustrated by Wallace, was very simple; consisting of just an orifice/small current path between electrodes. The modern version of the device can be applied in many areas, including medical instruments, for applications such as counting and analysis of blood cells, proteins, bacteria and prokaryotic cells (Kulp et al., 2004). It made possible the most common medical diagnostic test- the complete blood count, to be carried out for many samples in a very short period of time.

The first commercial counter, the “Coulter Counter Model A” incorporated novel components, like the invention of mercury manometer based metering system to tackle the challenge of accurately measuring the sample volume flowing through the orifice (Hogg and Cooley, 1964). The other essential element to the device was the electronic circuit for tracking individual events passing through the aperture. The change in electrical impedance due to passage of particles through the aperture is analyzed using pulse processing (Graham, 2003). Through this basic electronic circuit, the number of pulses that are detected can be correlated to the particle count, and the height (amplitude) of the pulse proportionally relates to the volume of the particle (Bull et al., 1965). In the early version of the device, an initial calibration was required to correlate the cell size to the threshold values on the oscilloscope to account for background noise level and cell type. Using the principle that larger cells produce high amplitude pulses in relation to smaller cells, various cell types are distinguished and characterized by analyzing the signal peaks. Wallace accurately demonstrated this method by determining the number of RBCs and cancer cells, based on the pulse information, from a sample solution. A lot of work published through the 1960's also looked at answering the challenges of particles simultaneously transiting through the aperture (Edmundson, 1966; Last and Smol, 2006). A major assumption made by impedance flow cytometers is that only one particle passes through the “sensing zone” at a time. The passage of more than one cell at a time causes an artifact known as coincidence. On an oscilloscope, this can be detected as a change in pulse height and/or width compared to a single cell pulse, for coincident passage. This can be minimized by reducing the aperture size or increasing dilution of the solution. A mathematical treatment was also proposed for coincidence correction. The mathematical expression obtained defines the count loss that occurs and can be factored into the data to give a more accurate result (Princen and Kwolek, 1965). Newer commercial devices contain threshold comparators that only allow pulses through that are equal or greater than the value predefined by the user for a cell type, thereby allowing processing of a heterogeneous cell population (Guo et al., 2012). Data can then be obtained on these pulse parameters (e.g., height and width of pulse), which can be converted to the required information such as cell size and distribution. With the help of advanced electronic circuitry, modern coulter counters employ digital signal processing. By means of automation, the data is displayed in a readily accessible manner to the user by tabulating the various morphological parameters.

Most early implementations of coulter counter use direct current resistance between the electrodes since that generates data, which is easy to process (Graham, 2013). Subsequently, patents were published to use high frequency AC currents coupled with low frequency or DC current (Wallace and Counter, 1966). The advantages of incorporating AC capacitance measurement is that it gives detailed information on the nature of the particle such as cell composition based on its dielectric properties (Holmes and Morgan, 2010). When the impedance measurement is made for two different frequencies, the device can distinguish between particles of identical size but different cellular contents. Byerly et al. demonstrated the use of AC technique to detect and count organisms by means of tracking the change in current as the particle passes through two electrodes (Byerly et al., 1975; Hoffman et al., 1981).

Coulter principle has been widely adopted across many industry and clinical applications since its conception. (Kubitschek, 1958). MS parker et al. made an attempt to use coulter counter to measure the swelling of bacterial spores during germination and growth based on size distribution. Since impedance based counting is a label-free technology, it is suitable for counting virtually any kind of cell, revealing the cell size and morphology (Parker and Barnes, 1967). In another instance, electronic impedance counting using coulter counter was used for standardization of yeast inoculum preparation for fungal susceptibility testing, and results were compared with culture colony counts. The results showed that coulter counter had great inter-laboratory reproducibility and correlated with the colony counts (Eng and Valensteini, 1989). However, most of the work using coulter counter was focused on hematology. Platelet counts using a coulter counter were reported on patients undergoing chemotherapy with acute leukemia and megaloblastic anemia (Bessman et al., 1982). Later, there were attempts to enumerate stem cells using coulter counter and it was observed that they provided reliable and reproducible results with CVs comparable to hemocytometer (Fernyhough et al., 2004). Simiele et al. proposed the use of coulter counter for measuring intracellular concentration of drugs. They determined the mean corpuscle volume using coulter counter method (Beckman Coutler Z2™) from HIV positive samples, which was then used to calculate the anti-HIV drug concentration. Traditionally the concentration of intracellular drug required sensitive instrumentation, such as mass spectroscopy coupled with liquid chromatography, due to very low concentration. The use of coulter counter provides significantly reliable and accurate way to quantify antiretroviral intracellular drug concentrations (Simiele et al., 2011).

**Figure 3:**
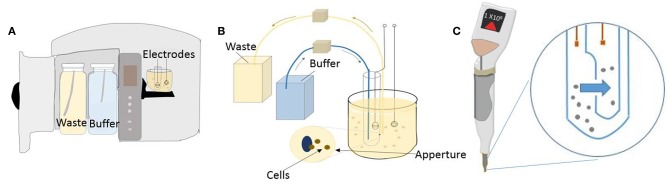
Coulter counter technology used in a benchtop and handheld device. **(A)** Schematic of a standard benchtop coulter counter. **(B)** Illustration of the mechanism of detection used in benchtop coulter counter. **(C)** Illustration showing Scepter handheld coulter counter and its working principle.

Since its early days in 1960's, technology in commercial impedance counters has evolved immensely. These modern instruments count tens of thousands of cells in just a few seconds and boast very low coefficient of variance. The dynamic range of the instrument- that is, the size of the particles that can be analyzed- depends on the aperture size and is central to the application. For example, benchtop Coulter Counter Z series™ By Beckman Coulter offers several aperture sizes to choose from, giving a range of 1 μm to 120 μm particles that can be analyzed. Hence the device can count and analyze various types of mammalian cells including blood cells, yet has the capabilities to work with bacterial or plant cells. Similarly, their another benchtop counter called coulter counter Multisizer™ is made to handle anything including industrial particles because its aperture tubes can handle particles up to 1,600 μm (Figure 3A). On the other hand, manufacturer's like EMD Millipore and ORFLO offer miniaturized impedance counters for research laboratories. EMD Millipores' pipette shaped handheld Scepter™ 2.0 aspirates sample through its disposable sensors and presents histograms on the counters screen display. Figures 3B,C show the coulter counting principle with an orifice and channel incorporated inside the scepter counter tip. More recently, relatively new companies like Izon Science™ are coming with innovative solutions like putting a nanopore hole in a plastic membrane which can mechanically open or close, instead of standard fixed aperture, thereby giving an even larger dynamic range. QNano Gold™, a device from Izon Science, were able to analyze sub-micrometer particles like viruses and micro-vesicles (van der Pol et al., 2013). Most certified clinical impedance based hematology analyzers, such as Sysmex XE 5000™, use multiple cell enumeration technologies in tandem to perform 5- part blood differential counts (lymphocytes, monocytes, granulocytes, eosinophils, basophils). They use dual impedance and optical methods to maximize instrument output (Paris et al., 2010). They supplement coulter principle with optical fluorescent based flow cytometry in their devices to ascertain multiple parameters. For instance, when performing platelet counts, abnormally large or fragmented platelets can skew data. The accuracy in reporting is increased by using fluorescent platelet count as a complimentary parameter to the impedance count. Although there are many commercial devices in the market that implement the impedance principle for cell enumeration, several attempts have been made to miniaturize these systems.

#### Microfluidic Impedance Analysis

More recently, advances in impedance cytometry have been achieved using cleanroom microfabrication protocols to miniaturize sensing and detection components through the introduction of microfluidic impedance analysis systems. When microfluidics was first introduced to cell counting/particle detection, its main goal was to bring portability, low cost, and simplicity (Sun and Morgan, 2010). It offers distinct advantages as they take less sample, have low power consumption, and offer point-of-care solutions (Cheng et al., 2009). Early impedance flow cytometry systems were only capable of making measurements in large sample volumes. Now, research in microfabricated impedance analysis is growing since it offers high sensitivity coupled with reduction in sample size. Microfabricating the impedance sensing technology alleviates some of the problems encountered by macro scale impedance flow cytometers such as the requirement of large sample volumes for analysis. Microfluidic impedance counter work on the same principle as the commercial coulter counter based devices, wherein there exists a pore in the microchannel which creates a change in electrical resistance when the cell/particle passes through it. This change can be processed by the amplification circuit and analyzed by the computer. For clinical applications that use impedance flow cytometry such as blood differential counts in hematology analysis, microfluidic chip based counting offers a way to make it cheap and available to remote locations without medical infrastructure. In addition, some devices employ an adjustable aperture thereby allowing larger range of particles and cell sizes to be detected (Rodriguez-Trujillo et al., 2008). The adjustable aperture in this context does not refer to a physical component, but to the spatial positioning of the cell within a fluid stream which was achieved using hydrodynamic focusing in 2 dimensions. The focalized sample volume acts as an aperture, which can be adapted to the particle size, as the sample flows between two electrodes and impedance is measured. Such a system supports a wider threshold of particle dimensions to be detected since the aperture size can be controlled by varying the focusing flows. Such sub-micrometer scale coulter counters hold important applications in DNA sequencing. With the help of modern microfabrication techniques, it is possible to fabricate sub-micrometer channels and apertures for counting and detecting particles, as small as single DNA/RNA molecules (Spinney et al., 2012).

**Figure 4:**
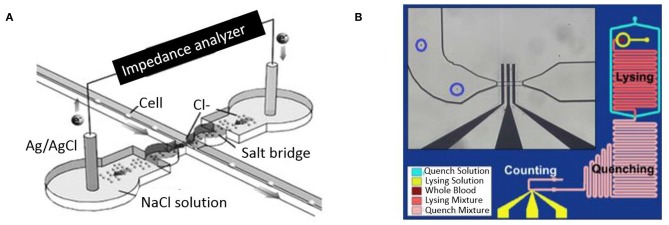
Microfluidic impedance cytometers for cell detection. (A) A micro-Coulter system employing two Ag/AgCl electrodes through a salt bridge (Chun et al., 2005). Reprinted with permission from Analytical chemistry. (B) A Microfluidic platform lysing RBCs from whole blood before performing a cell count (Hassan et al., 2015). This work is licensed under the Creative Commons Attribution 4.0 International License.

One of the earliest examples of a microfluidic coulter counter employed DC voltage to count living cells (Larsen et al., 1997). Figure 4A shows DC impedance sensing accomplished using salt-bridge electrodes across a microchannel. The resulting micro-device was used for evaluating human blood cells and achieved cell counting as well as measurement of cell velocity up to 100 mm/s (Chun et al., 2005). A different approach utilizing AC frequencies instead has also been used to quantify and characterize cells in a microfluidic device. This was first elucidated by Ayliffe et al. who used single cell impedance spectrum discrimination for cell analysis (Ayliffe et al., 1999). The method involves AC current that is applied across passing cells which causes polarization due to accumulation of charges at the interface of the cellular membrane and the suspension medium. Such application of impedance cytometry to micro/nano-fluidic channels provides higher sensitivity (Zhang et al., 2009). Figure 4B shows a microfluidic device where RBCs are lysed on the chip and leukocytes in whole blood sample are counted. Hence the impedance measurements made at such wide frequency ranges provide information on cell size, membrane capacitance, and cytoplasm conductivity along with cell concentration (Petchakup et al., 2017). The wide bandwidth impedance signal processing and analysis from AC microfluidic devices requires sensors for probing impedance measurements at multiple frequencies. Fuller et al. used discrete mixers, filters and direct digital signal synthesis circuits integrated with microfluidic chips for measuring impedance at different frequencies (Fuller et al., 2000).

Mikael Evander et al. used impedance spectroscopy at multiple frequencies ranging from 280 kHz to 4 MHz for determination and analysis of platelets from blood samples (Evande et al., 2013). Sohn et al. introduced a significant microfluidic device utilizing flow cytometry based on AC capacitance in an integrated microfluidic chip for measuring biologicals cells. Cells were characterized by distinct peak in signals as they flow past electrodes at a fixed frequency of 1 kHz frequency (Sohn et al., 2000). Later, from the same group, a rapid and quantitative measurement of nanoscale colloids was demonstrated by successfully fabricating a counter using a quartz substrate (Saleh and Sohn, 2001). The application of multi-frequency microfluidic counters is found across several domains including stem cell differentiation (Bagnaninchi and Drummond, 2011), white blood cell differential count (Holmes et al., 2009), cell sensing for drug delivery (Ramasamy et al., 2014), enumeration of CD4+/CD8+ from whole blood samples (Watkins et al., 2013), and complete blood cell counts at point-of-care settings (Hassan et al., 2015). Despite the many advantages of microfluidic technologies, challenges like low-throughput makes it unsuitable for measuring and processing large volume of samples such as rare cells within a heterogeneous background population of billions of cells in body fluids. Multiple frequency AC spectroscopy also requires impedance analyzers and lock-in amplifiers to acquire and analyze impedance signals along with bulky fluid pumps for introducing the sample through the microfluidic chip which makes it hard to achieve portability.

#### Electrode Design and Particle Focusing

Various electrode design and configurations have been investigated for use as microfluidic impedance sensors. Studying electrode geometry is important to achieve better detection sensitivity because of variability in the electric field distribution created by electrode design as cells go through their trajectories and create impedance signals. For higher sensitivity, the size of the electrodes has to be comparable to the size of cells. In order to facilitate this miniaturization, microfabrication techniques have been used extensively (McDonald et al., 2000). The material chosen for electrode fabrication depends on its electrochemical properties and manufacturability. Gold (Au) is commonly used because it is electrochemically stable and biologically inert, but other materials like Silver (Ag), Platinum (Pt), Nickel (Ni), and even liquid electrodes made by inserting Silver/Silver Chloride (Ag/AgCl) wires in conductive electrolyte solution have also been demonstrated (Shrirao et al., 2018). Although several variations of electrode configurations exist, the two most common configurations adopted in microfluidic devices are the co-planar and parallel electrode arrangements.

**Figure 5:**
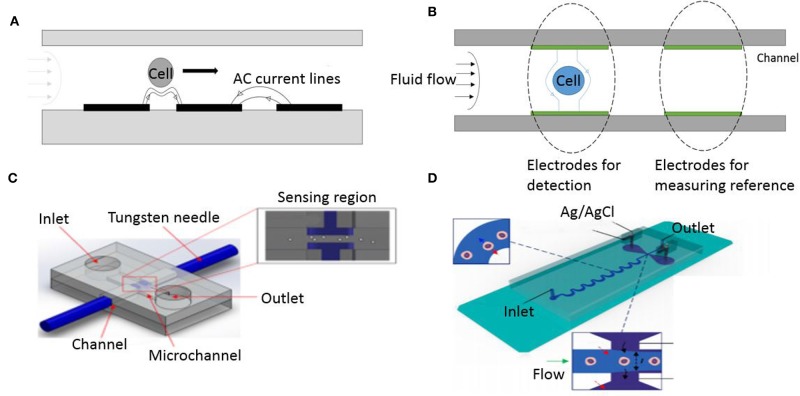
Different electrode geometry configuration. **(A)** Cell flowing through co-planar electrodes. **(B)** Illustration of a cell flowing through a parallel electrode configuration inside a microfluidic channel. **(C)** Schematic diagram of needle electrodes (Mansor et al., 2017). This work is licensed under the Creative Commons Attribution 4.0 International License. **(D)** Liquid electrodes made of Ag/Agcl (Tang et al., 2017). Reprinted with permission from Analytical Chemistry.

Gawad et al. introduced co-planar electrodes and devised a microfluidic system to measure the spectral impedance of individual cells or particles at multiple frequencies (Gawad et al., 2001). They achieved significant advancement in microfluidic impedance flow cytometry by fabricating co-planar electrodes on the bottom of a microfluidic channel as shown in Figure 5A. This device used differential signals to record each particles impedance with the suspension media as a reference. AC voltage was supplied to the electrodes which resulted in a non-uniform electric field. Differential impedance variation at multiple frequencies ranging from 100 kHz to 10 MHz, is measured as cell passes through two successive segments. The device successfully discriminated between erythrocytes and beads based on the impedance spectrum. Their co-planar electrode structure provided the advantage of deriving cells speed and height as it traverses through the electrodes because the distance between the electrodes and time separating the spikes on plot are known. They are also attractive because of ease of microfabrication yielding miniaturized low cost devices. But the major disadvantage of this arrangement is that identical cells traversing at different heights in the channel give different impedance signals (Spencer and Morgan, 2011).

The other most common electrode configuration used in impedance flow cytometry systems is the parallel overlap arrangement. Cheung et al. first fabricated parallel facing microelectrodes in a fluidic channel (Cheung et al., 2005). The device consisted of a pair of measurement electrodes and an additional pair of reference electrodes placed symmetrically, as shown in Figure 5B, to allow simultaneously measurements at multiple frequencies. Their device successfully differentiated between beads and RBC's as well as fixed RBC's (defined as RBC's treated with glutaraldehyde) and normal RBC's. The cell velocity was limited to 10 mm/s to avoid shear stress deformation on RBC's flowing through the channel. This technique involves fabricating electrodes on two identical wafers which then need to be thermally bonded to create closed channels that have electrodes aligned face-to-face. To overcome the complicated method of fabricating electrodes in microfluidic chip, other alternatives have been made such as using reusable microneedles made of tungsten (Mansor et al., 2017), shown in Figure 5C. Mansor et al. used the device to detect cell concentrations of yeasts at high frequency ranges, between 100 KHz and 5 MHz. This makes the device low-cost since it does not require expensive fabrication for patterning the electrode or probe on the substrate. In addition, other research groups have also explored using electrodes deposited on a SU-8 coated printed circuit board as a low cost platform- for developing countries. Guo et al. made an impedance cytometer built on a PCB for cancer cell detection and enumeration (Guo et al., 2014). To address the issue of low throughput faced by coplanar and parallel microelectrodes, Tang et al. demonstrated the use of liquid electrodes (Tang et al., 2017). This method improves detection sensitivity while maintaining high flow rate of samples through the channel (Figure 5D). Their device uses a pair of perpendicular positioned liquid electrodes to the channel flow and can distinguish cancer cells that are spiked in a suspension of WBCs.

In order to mitigate the influence of positional alignment as the sample passes through electrodes, several fluid flow focusing techniques have been proposed. This ensures that the cells flow in a single stream in the interrogation region. Holmes et al. used dielectrophoresis (DEP) to focus particles within the flowing fluid and demonstrated it by counting fluorescent latex particles at rate of 250 particles/s (Holmes et al., 2006). Similarly, Mernier et al. made use of lateral focusing to focus particles in the middle of the channel to reduce variation of measurements. The device uses DEP focusing using electrodes with large surface area for easy fabrication while allowing measurement at lower frequencies (Mernier et al., 2012). However, the most common method employed to focus particles to the middle of the channel and reduce variation in measurements is by hydrodynamic focusing. A novel 3D hydrodynamic focusing was achieved by using sheath fluid on both vertical and horizontal direction (Testa et al., 2015). This provides increased detection sensitivity as it creates a single file of particle stream by varying the sheath to sample flow ratio. 2D hydrodynamic focusing to spatially confine particles in a microchannel was developed to identify both beads and bacterial cells for cytometric detection and quantification (Canjun Mu et al., 2011). A different sheath-less approach relayed on staggered channels design consisting of curved and straight sections that combine to order particles in a single stream (Oakey et al., 2010). They showed inertial focusing in straight channels and curved channels with varying modes of symmetry. High aspect ratio channels focus beads to two lateral positions while asymmetrically curved channels focus beads to vertical positions. Combining these channels in series biases the entire particle population to one half of the channel where they are focused to a single vertical streamline within the straight channel. Others exploit signal processing methods to reduce the positional dependence issue without the need for particle focusing (Erricoa et al., 2017). Table 2 summarizes the various fluid focusing methods commonly employed in microfluidic devices.

**Table 2:** Summary of fluid focusing methods in microfluidic cytometry.

**Focusing Method**	**Principle**	**Advantage**	**Disadvantage**
Sheath flow	2D and 3D Hydrodynamic focusing Ligler and Kim, 2010; Golden et al., 2012	Fabrication of fluidic channels without the need for active electrical circuitry	Production of large amounts of waste sheath buffer fluid Need for multichannel fluidic pumps to precisely position cells within the fluid Increased cell concentrations can result in the loss of a focused single cell stream resulting in erroneous cell counts
Sheath-less flow	Acoustic focusing Piyasena et al., 2012	High volumetric throughput Precise Spatial Positioning within 3D sheath flow	Requires integration of piezoelectric devices to generate acoustic waves
	Dielectrophoretic focusing Yu et al., 2005	Similar efficiency to acoustic focusing	Requires electrode integration within the channel Requires sample buffer conductivity to be adjusted Depends on particle polarizability
	Inertial focusing Gou et al., 2018	Passive method not requiring external driving power	Diminished performance at high cell concentrations similar to hydrodynamic focusing Results in pressure variations and consequently the shear stresses
	Magnetic focusing Zeng et al., 2012	Precise spatial positioning can be achieved by extrinsic magnetic bead labeling	Few biologicals particles are diamagnetic like erythrocytes and platelets Other cell types need to be tagged/ labeled using magnetic beads Requires the integration of strong magnets to produce intense field gradients

Microfluidic impedance flow cytometry has evolved due to developments in particle focusing and microfabrication of electrodes integrated within the fluidic channels. The high sensitivity of impedance detection has allowed detection of particles of submicron sizes (Petchakup et al., 2017). Impedance approach can offer a cost effective and high throughput solution for cell detection without the need for biomarkers to label cells.

### Optical Cytometry

Developments within the fields of laser technology, signal processing, antibody production, and fluorochrome chemistry led to the advent of optical based detection methods. The first optical cytometer was developed approximately two decades after the advent of coulter counter (Mittag and Tarnok, 2011). The initial instrument was large and cumbersome, making it difficult to operate, hence they were principally used in research laboratories. However, the benefits of optical flow cytometers over coulter counters in terms of their multi-parametric capabilities were soon realized and they were adopted more commercially. Current state-of-art optical flow cytometers are capable of analyzing up to 20 parameters (forward and side scatter and 12 fluorescence channels) on each cell at very high rates (Chattopadhyay and Roederer, 2012). Advancement in automation and robotics has helped improve efficacy of these devices. Flow cytometry is employed in cell analysis by suspending them in a stream of fluid and passing through an optical detection apparatus. The device is now routinely used by healthcare professionals in clinical diagnostic application (Pedreira et al., 2013). Modern optical flow cytometers perform several features, such as cell sorting, making them indispensable in clinical practice.

Technological developments have enabled the creation of FACS systems using flow cytometry. The first fluorescently labeled antibodies using Fluorescein Isothiocyanate (FITC) was developed by Albert Coons in 1941, which led to the creation of FACS by Herzenberg and commercialized by Becton Dickinson in 1974 (Moon et al., 2010; Adan et al., 2016).

**Figure 6:**
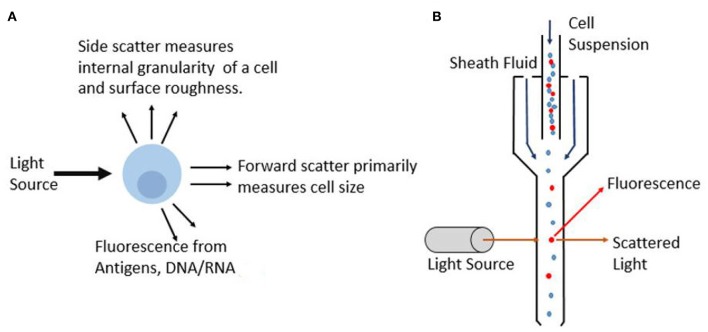
Principle of optical flow cytometry. **(A)** As incident light beam hits the cell, different parameters such as extinction, scatter and fluorescence are measured and this interaction provides information on optical properties and composition of the cell. **(B)** Schematic showing the particles lined in a single stream using sheath fluid as they interact with the laser light which gets collected by detectors.

Flow cytometry on cells quantifies the optical and fluorescence characteristics of individual cells in flowing liquid as they pass through an optical and fluorescence light path. The fluidic setup is comprised of a central fluid stream in which the sample is contained, surrounded by a sheath fluid which provides hydrodynamic focusing to create a single stream of particles. This is essential so that only a single cell can pass through the light path at a time to prevent errors in cell counts. Flow cytometers are now available with more customizable parts that enable optimized operation based on the cells being studied. For example, various nozzle sizes are available to control the pressure and the shear stress on cells helping to improve and preserve viability in cell sorting applications (Graves et al., 2002). As each particle subsequently passes through the light beams, which are commonly generated by lasers, the scattered light provides data on the particles. In most flow cytometer instruments, several lasers are used that produce various wavelengths ranging from Ultraviolet (UV) to far red (Telford, 2004). These lasers are typically quite compact as they employ solid-state electronics. Moreover, with advances in technology, these lasers offer superior beam quality and minimum laser noise. Light that is scattered in the forward direction is picked up by photomultiplier tube (PMT) and is referred to as forward scatter (FSC), and it primarily provides information on the size of the particle. Whereas, light that is scattered in an orthogonal direction to the initial laser beam is referred to as side scatter (SSC), and this provides information on cell morphology and internal cell complexity. Figure 6A illustrates the information that can be gathered as an incident light beam hits the particle. Based on the detection of the light from the particle, different parameters can be obtained.

Cells can be quantified not only based on forward scatter and side scatter, but also on the basis of a fluorescence light (FL) from the fluorochromes used for the detection of target cells (Brown and Wittwer, 2000). Various optical components are used in flow cytometer to direct photons to the respective photomultiplying tubes while minimizing spectral overlap. Fluorochromes are generally chosen such that they satisfy a few conditions. The chosen fluorochromes should minimize spectral overlap and should be very bright. In addition, the biological antibody heterogeneity must be considered, and the brightest flourochromes must be used for dim antibodies and vice versa to maximize detection ability. Figure 6B shows the working principle of commercial flow cytometer. The suspended cells are focused in the fluidics system to ensure that cells travel through the center of the channel with a uniform velocity. Sheath fluid surrounds the cell suspension to align them in a single stream as it passes through the detection region. Conventional systems use the optical signals based on the scattered light and emitted fluorescence to characterize individual cells or particles based on several parameters in a heterogeneous population. Another alternative that is gaining popularity is the use of quantum dot probes. Unlike commonly used organic based fluorochrome, such as FITC, quantum dots have a broad excitation spectra and are able to remain fluorescent under constant illumination without photobleaching which results in greater precision and flexibility (Buranda et al., 2011). Recently developed mass cytometry, which is a variation of flow cytometry, works by labeling cells with metal based cell markers as opposed to fluorochromes. This can provide more than 40 parameters on the cell and is particularly useful for single cell analysis (Guo et al., 2017). This augments the ability of cytometer to evaluate complex cellular systems such as enabling biological research to immune cell responses.

Flow cytometry is used in various cell enumeration applications based on the detection of the membrane, cytoplasmic and nuclear antigens (Lan et al., 1996). Additionally, whole cells and cellular components such as organelles, nuclei, DNA, RNA, chromosomes, cytokines, hormones, and protein content can also be investigated by flow cytometry (Garner et al., 1983; Jung et al., 1993). Flow cytometry has also been exploited for drug detection in order to investigate cell uptake in several chemotherapeutic delivery systems. Nano-particles such as cell derived vesicles and other vectors are being used as models for carrying drugs to target cells (Goh et al., 2018; Tapeinos et al., 2019). In addition, flow cytometry is used for the detection of microbial contamination in food supplies by gathering information on the physiological state of microbes to detect and identify specific forms of contamination. Contrary to colony counting method which can take long time based on growth of organisms on agar plate, cell counting and viability assessment using flow cytometry is rapid, taking approximately 5 min for processing each sample and providing a much higher level of accuracy. In addition to this, flow cytometry can be used to assess the impact of various food processing treatment such as pressure processing, thermal treatments and UV radiation (Comas-Riu and Rius, 2009).

Modern optical flow cytometers have evolved immensely to be more user-friendly through the use of intuitive digital interfaces and simpler operation protocols. BD Accuri™ c6 plus is a great example of a device used widely in research for studying cell viability, absolute cell counts and monitor cell proliferation. The device uses peristaltic pump which drives the fluidics such as sheath fluid (composed of distilled water) and sample. It uses 4 fluorescence detectors for the detection of common fluorochromes such as FITC among others, and 2 scatter detectors. EMD Millipore also offers a benchtop optical flow cytometer called Guava easyCyte™. Their use of a novel microcapillary method eliminates the need for sheath fluid, hence making it more compact than Accuri™ c6. The easyCyte™ can provide enumeration of cell population using small sample volume. Attune NxT™ is another device available in the market from ThermoFisher scientific that utilizes acoustophoresis instead of hydrodynamic focusing to position cells along the flow channel. Using acoustic method, the device can achieve high throughput to perform analysis. Such commercial devices offer great precision and sensitivity for cell population enumeration. But the initial investment and large size can be challenge for implementation in remote, low resource deployment. To tackle this bottleneck, researchers have attempted to integrate optical components onto lab-on-a-chip devices for inexpensive point-of-care settings.

#### Microfluidic Optical Flow Counting System

Fueled by the need to reduce size and cost of flow cytometers, significant effort has been made to miniaturize and make point-of-care optical flow cytometry systems based on microfluidic chips. At the time that impedance measurement systems exploited the potential of microfluidics for low-cost cytometry analysis (as described in previous sections), researchers also focused on developing microfluidic cytometer with optical detection systems. They are similar in principle to conventional flow cytometers, in that they rely on optical system and sensors for particle detection. To achieve microfluidic optical cytometry systems, several necessary technological innovations have been proposed toward microfabrication of fluidics system for introduction of samples inside the miniaturized channel and integration of optical excitation and detection system (Cho et al., 2010; Verellen et al., 2018). The application of these devices is found across several domains such as CD4+ T cells, RBCs, and platelets counting (Evande et al., 2013; Watkins et al., 2013)

The mechanisms used in conventional systems for fluid flow, focusing, optical illumination and detection need to be redefined to suit microfluidic platforms. A major component of microfluidic flow cytometry is the single stream focusing of cells in the channel to avoid coincidence of multiple cells passing through the detection zone. This is accomplished through various flow focusing methods such as hydrodynamic focusing using sheath flow, dielectrophoresis and inertial microfluidics (Gong et al., 2018). Waveguides or optical fiber are most commonly employed to route light to the chip based on total internal reflection. Various methods to deliver and detect the light beam have been explored using different fabrication techniques (Mohan et al., 2017). One of the most important components of the detection system is the photodetector and its integration into the device is key to producing portable devices (Martini et al., 2012). Measurements from these photodetectors are very sensitive to misalignment.

Several materials and optical configurations have been studied for development of sensitive yet robust microdevices. Sobek et al. achieved fabrication of flow chambers by bonding together two etched silicon wafer substrates using borophosphosilicate glass (Sobek et al., 1993). This assembly allowed them to integrate optical waveguides into the flow duct for collecting scattered laser light. By employing waveguides, they were able to couple laser light in and out of the chip with minimum propagation loss. Chabinyc et al. made a noteworthy device using rapid prototyping for fabrication of Polydimethylsiloxane (PDMS) microfluidic channels (Chabinyc et al., 2001). They integrated a micro-avalanche photodetector in the PDMS channel for fluorescent detection, without the need for a detection waveguide. This eliminates the need to have transfer optics such as waveguides and optical fibers, since the detector was built into the microfluidic system. The device used an LED as the excitation source which was delivered using optical fiber placed inside PDMS and successfully detected proteins labeled with fluorescein. Pamme et al. performed the counting of C–reactive protein using measurement of laser light scattering at two different angles (Pamme et al., 2003). Localized optical detection system in the chip is imperative for scatter measurements with low coefficient of variation (CV). This will reduce the effect of scattering from debris in the sample fluid. The CV is an important parameter for signal quality of a flow cytometer. They utilized Poly (methyl methacrylate) or PMMA with 50 μm deep inlet and outlet channels for sample flow to minimize the internal reflections occurring at the PMMA/air interface. The fluid was flown through the channels from the reservoir using negative pressure generated by a syringe pump. The two fibers were mounted externally above the flow channel at two different angles to collect scattering signals which was then sent to detection system consisting of PMT's. This allowed for particle detection in the range of 2–9 μm. Wang et al. demonstrated scatter measurements by integrating all optical elements in SU-8 polymer. Rapid prototyping using SU-8 polymer permitted integration of waveguides for incident and scattered laser into the channel, all of which could be made in a moderately short period of time (Wang et al., 2004). While testing scatter signals from beads, a CV of 29.7% was observed for beads of diameter 9.1 μm. This is relatively large compared to conventional flow cytometers and can be attributed to the use of bulk optics for the optical measurements resulting in alignment problems and shock stability. The use of simple waveguides holds the risk of contamination from stray light due to substantial degree of noise as these accept light over their entire numerical aperture. Also, the device uses sheath flow from just two lateral sides and not from the top and bottom, as opposed to 3D fluid routing where the sample is sheathed and focused from all sides. This is increasingly important as the sample size decreases and is essential to reduce variation. To address this issue, Fan et al. designed a high-throughput, 3-D microfluidic device to ensure that the particle gets uniform lateral and vertical focusing so that they receive equal excitation laser intensity which was detected on a CMOS sensor (Fan et al., 2013). Multiple sample and sheath flow channels as well as micro-ball lens array were fabricated in 3D PDMS layers of the microfluidic chip. Using parallel analysis, the device can achieve a detection rate of 188,800 cells/s which is much higher than standard commercial device and suitable for detection/enumeration of rare cells, such as CTCs, with a background of billions of other cells. Such simplified and integrated chips show promise in presenting multiple parameters with low CV's. In addition, a system with 3D flow focusing and integrated collection light system consisting of input and output waveguides for FSC and SSC detection was used by Godin et al. to create a microfluidic cytometer (Godin and Lo, 2010). The scattered light is collected at two different angles at the interrogation location using unique tapered waveguides and uses PMT and photodiode for laser detection. The device offered good CV due to a 10-fold decrease in signal to noise ration from its tapered waveguides built into the PDMS. This device has a capability to characterize bead populations at throughput of up to 800 particles/s. Similar integrated waveguides, made of liquid core using immersion oil (refractive index *n* = 1.515) as opposed to the traditional solid waveguides used in optical chip based cytometers was also later demonstrated (Fei et al., 2012). These waveguides are built into the PDMS chip by filling selected microfluidic channels with specific optical fluids, while other channels are used for delivering samples. The cells get excited and emit fluorescence which is collected by the two detection waveguides. The intensity changes are obtained as distinct peaks used for enumerating cells. The signal is collected by the waveguide and coupled with CCD camera for detection. The cytometry device is able to count cells in a sample at a rate of 50 cells/s.

**Figure 7:**
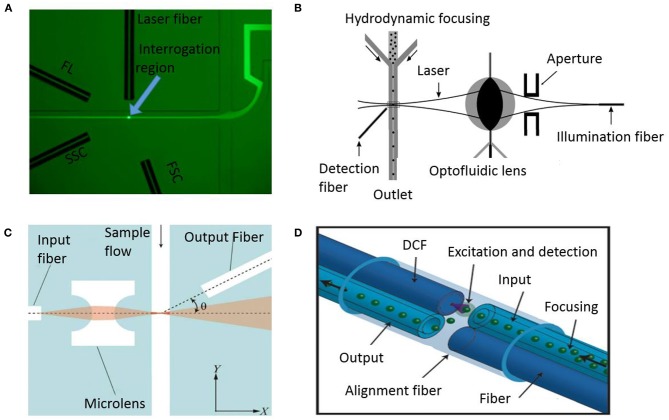
Microfluidic optical flow cytometers. **(A)** A microscopic image indicating hydrodynamic focusing and the arrangement of the optical fibers in a flow cytometry chip (Mao et al., 2012). FL, FSC, and SSC stand for fluorescence light, forward scatter, and side scatter, respectively. Reprinted with permission from Biomicrofluidics. **(B)** Schematic configuration of a microfluidic flow cytometer using an optofluidic lens (Song et al., 2011). This work is licensed under the Creative Commons Attribution 4.0 International License. **(C)** System showing integrated optics comprising of fibers and microlens in PDMS (Zhao et al., 2016). Reprinted with permission from Biomicrofluidics. **(D)** Schematic of a fiber-based micro-flow cytometer with an integrated detection micro-chamber encased in a double clad fiber (DCF) (Etcheverry et al., 2017). This work is licensed under the Creative Commons Attribution 4.0 International License.

More recently, Schafer et al. enumerated cancer cells on their microfluidic device and compared it to commercial systems (Schafer et al., 2009). The device was made of glass using femtosecond laser ablation and anodic bonding. The development of glass microfluidics helps in overcoming the optical limitations of PDMS while still creating channels and integrating optical fibers to contact fluid through their unique single step fabrication method. Using an inexpensive photodiode, their device offers sensitive optical detection with low power consumption. However, the detection parameters of these microfluidic cytometry devices do not achieve the multiparametric detection capabilities of benchtop counterparts. To address this issue, Mao et al. designed an integrated device with 3D hydrodynamic focusing combined with optical fibers (Figure 7A) to facilitate on-chip detection using all three optical output signals: Forward scatter, side scatter and fluorescence (Mao et al., 2012). The optical fiber module consisted of one input fiber and three detection fibers integrated in chip. Laser coupled to the input fiber is incident perpendicular on a single stream of cells at the detection point. The three detection fibers were arranged around the fluidic channel at different angles to gather signals simultaneously. Then, the repeatability was tested by measuring CV for the chip and comparable results were obtained to conventional flow cytometers with just slightly larger values. The device has the capability to detect particles with a throughput of 685 particles/s. In addition, others have considered addressing the challenges of integrating optical components, such as lenses, and scattering due to external optical elements. A device made using optofluidic lens was developed by Song et al. to reduce scattering and improve accuracy (Song et al., 2011). Figure 7B shows the optofluidic lens developed to focus laser from the optical fiber tip to the detection point in the channel. Low CV was achieved in particle detection compared to previous cytometers. A more integrated optical system in the microfluidic channels was developed by Zhao et al. Their optical excitation and collection fibers, and microlens were fabricated inside the PDMS channels which allowed them to achieve significant size reduction (Zhao et al., 2016). Figure 7C shows how all the optical components are on the same plane thereby allowing the microlens to focus laser light on a narrow region of sample flow channel while the detection fibers collect the scatter information. Another unique approach involving micro-chamber cytometer using an all-silica fiber for the transport of cells and collection of scattered and fluorescent laser inside the capillary housing was reported (Etcheverry et al., 2017). The system integrates transport of cells and detection of optical signal in the same micro-chamber making it an attractive point-of-care system (Figure 7D).

Microfluidic optical flow cytometers have brought accurate and high throughput way to enumerate and characterize cells. Advancements in microfabrication methods have allowed integration of optics and detection system to the chip. They are shown to be capable of detecting and quantifying cells of different size better than impedance based methods by labeling them with fluorescent markers. However, to make the device more practical, they need to match the low CV's and multiparametric capabilities of benchtop flow cytometers. Most of the attempts at optical microfluidic flow cytometry involve using conventional hardware such as lasers, detectors, fluid pumps, high power light source and electronics while just miniaturizing the microfluidic chip. The main disadvantages of using bulk optics for microfluidic systems are the size, susceptibility to shock, and alignment problems which prevents it from being truly portable.

### Image-Based Cytometry

With the advent of computers and rise in their processing capabilities in the last few decades, along with the emergence of inexpensive high-quality camera sensors, there has been a revival in image based analysis for cell counting. This is due to the advantages it provides- easier sample preparation and handling, economical, and visual information on the morphology of the cell (Lanigan et al., 1993). For that reason, there has been a rise on computer based image cytometry fueled by development of robust machine learning and image analysis algorithms. A software can now be trained to count cells in a sample, hence automating the mundane task and also making the process faster and consistent compared to tedious visual inspection. Image cytometry not only provides a platform for obtaining high-quality data, but also allows for inspection of data through robust software. Quantitative image analysis performed by computer is able to detect features not detectable by human observer. For example, the software can also pick up on other small features, such as increase in nucleus size, which are not noticeable otherwise. This allows label free detection between phases of the cell cycle, which is not possible using the conventional hemocytometer approach (Blasi et al., 2016).

Image based cytometry can broadly be categorized to static image cytometry and kinetic image processing performed on high resolution images from flow cytometers. Firstly, we will be looking into static image cytometry within the context of image analysis. Unlike optical flow cytometry systems, image based cytometry devices provide the advantage of identifying each cell using real images from the heterogeneous population. Most image analysis systems, such as Life Technologies^TM^ Countess^R^, use disposable slides with appropriately stained samples, using dyes such as Trypan blue, which the system images onto a digital camera. The device is categorized as semi-automatic because the user is responsible for performing the appropriate staining protocol to optimize the reading. Such a system may need an adjustment in staining to obtain a better resolution of cells from the background image, as captured by the auto-focus camera. High Trypan blue concentration in the sample can lead to background noise and interfere with the ability of the instrument to correctly distinguish cells. However, some image cytometers, such as the Vi cell XR by Beckman Coulter allow user to load several samples simultaneously (up to 9) and perform automated analysis. This eliminates the need for the analyst to perform staining on the samples; thereby making the sample preparation steps less time consuming.

**Figure 8:**
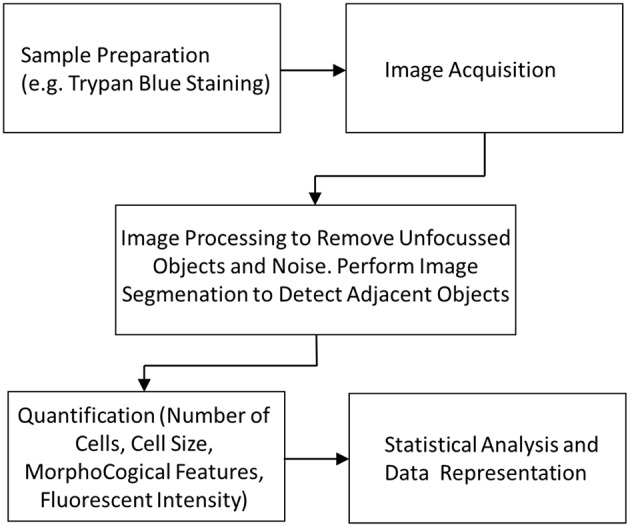
Image cytometry workflow showing the different stages from sample preparation to data analysis.

For the successful analysis of a sample it is important to have fast and accurate software which can gather data from the captured images. Figure 8 shows the workflow of steps required to gather data from a device using image-based cytometry. As described earlier, sample preparation protocols form the primary step in any image cytometry system. Appropriate selective staining with the right concentration of dye to perform the analysis is crucial. This allows for visualization of cells by the camera and get better resolution of cells from the background. This is followed by image acquisition by the system using bright field or fluorescence microscopy. This is followed by segmentation, also known as object identification, which is the most important step in the process for determining accurate cell measurements. Segmentation algorithm has to detect adjoined cell aggregates in a biological sample. Histological images of cells are difficult to analyze due to image artifacts that are introduced due to multistep preparation process as well as variation in batches due to different microscopy and staining steps. The technique must be able to extract information from images by combining local and global properties that are characterized by different pixel classes in the continuous boundary. Several methods were developed to accurately address cluster segmentation. Previous work by Sonal Kothari et al. described an edge based segmentation method that can segment complex clusters with reasonable accuracy (Kothari et al., 2009). Recently, deep learning methods are being applied to medical image analysis (Litjens et al., 2017). The method is inspired by neurons in the brain and involves creating artificial neural network that can transform input data to output. This is achieved by training the computer with annotated images and improving performance iteratively by using large variety of samples (Gupta et al., 2018). This emerging field presents exciting growth in image analysis as it has great power in improving the accuracy in cell detection and segmentation. Xie et al. successfully used neural networks for performing T-cell counts (Xie et al., 2018). Most commercial devices, which rely on static imaging, have built-in software modules which perform the segmentation to accurately identify and analyze individual cells within smaller aggregates. They use intensity peaks from the fluorescent stain to segment objects relative to the background intensity levels. This information from the images is further used for quantification, cell cycle analysis and other assays. The results can then be used for statistical data analysis and represented graphically for better validation.

In contrast, research in kinetic image flow cytometry offers an alternative to conventional flow cytometry through high-resolution imaging or microscopy of single cells in flowing environments. Essentially, the goal of such systems is to perform live cell imaging in a sample suspension using powerful image segmentation and feature extraction, as described previously, while combining features of flow cytometry. Hence, it allows evaluation of morphological and fluorescent data on single cell in a heterogeneous population. The limitation of static image cytometry due to challenges in image segmentation as described earlier has little effect on kinetic imaging flow cytometry as the cells are in suspension and interrogated on an individual basis. This progress was mostly due to emergence of high-speed cameras to image large number of cells per acquisition frame. For instance, McKenna et al. developed a device that utilized a parallel microchannel network along with confocal microscopy for rare cell detection (McKenna et al., 2011). The device achieved a throughput of 10^3^ cells/s. A fluorescent labeled approach that uses novel techniques to offer pixel readout rates much higher than CCD's was reported. The method was used to perform fluorescent imaging of human breast carcinoma. Meanwhile, similar imaging flow cytometry systems were also used for CTC detection as a more sensitive and reliable approach. Liu et al. elucidated a method to study morphological properties of cancer cells using imaging flow cytometry (Liu et al., 2016). After DAPI staining (a blue-fluorescent DNA stain) and antibody labeling, measurements of a new parameter called karyoplasmic ratio were performed. This is defined as the ratio of the area of the nucleus to the area of the cytoplasm. Using this method, they were able to conclude that CTCs had higher karyoplasmic ratio compared to healthy cells. Rane et al. presented a microfluidic imaging flow cytometer without the need of sheath fluid by using inertial focusing (Rane et al., 2017). This device can perform fluorescence bright field and dark field images for analysis of cell suspensions while still giving high throughputs in excess of 50,000 cells/s. More recent developments and research on image cytometers have been focused on introducing machine learning and interpretation capability to the devices. Using advanced machine learning systems, image cytometers are capable of analyzing and interpreting cells in a large dataset. This emerging technology was used by Heo et al. to acquire rich information from single cell images for enumeration and analysis (Heo et al., 2017). A real time image processing system called R-MOD (Real-time Moving Object Detector) was developed to analyze images from image flow cytometry. Using machine learning to image sequences obtained by CMOS camera, the system was able to achieve classification and cell counts. Such real time analysis ensures that huge amount of data in the form of acquired images does not need to be stored in memory for post-processing thereby addressing the computational challenges of image cytometry.

Currently, several commercial image-based cytometers are available for research and clinical use. Multi-parametric data is obtained by applying powerful image segmentation and feature extraction algorithms to raw data. Modern image cytometers combine the power of fluorescent detection with imaging capabilities as they are highly complementary, allowing multiple parameters to be measured for individual cells. Vast array of fluorescent probes and stains makes it possible to measure spatial distribution, intensities of markers on cells, as well as general morphological quantities to generate data on each cell. Simple cell counters like TC-20^TM^ by Bio-rad Laboratories claim to count mammalian cells in <30 s. It uses light microscopy with auto-focus that analyses multiple focus planes, along with a cell counting algorithm that is able to identify cells and exclude debris thereby calculating the total cell count. These kind of devices eliminate the need to dilute the solution (process a concentration range of 5 × 10^4^-1 × 10^7^ cells/mL), as usually done in hemocytometer, and reduce the error associated with sample dilutions. NC-3000™ by NucleoCounter utilizes fluorescent imaging to characterize cell properties. This is achieved by exploiting fluorescent dyes with affinity for DNA to generate images which are otherwise missed by light microscopy. Other devices such as FlowSight® Imaging Flow Cytometer combine flow cytometry capabilities with imaging and simultaneously produce dark field (side scatter), bright field and fluorescent images of each cell.

Image cytometry can measure information at single cell resolution. The method provides rich morphological data and overcomes the lack of this information from electrical impedance and optical flow cytometers. Specialty applications where specific information is required without destroying the cell, such as assays studying cell-cell interaction or morphological data make image cytometer ideal for use. The primary bottleneck of using this technology in clinical applications is the computational challenges in handling large volumes of image data that need to be processed in real time (Nitta et al., 2018).

## Conclusion

Tremendous progress has been made in each paradigm of cell enumeration. Several industry, clinical and research applications leverage the use of impedance, optical, or image analysis systems for cell counting and characterization. Each method provides advantages that need to be considered while keeping in mind the cost, throughput, accuracy, and portability of the device.

Cell analysis using a hemocytometer has been widely used for over a century, primarily due to its low cost and portability. The device is used by clinics to analyze blood samples and as a common tool in research laboratories around the world. However, due to the cumbersome process of manual counting, there was a strong push to develop automatic cell counting technologies which yielded high throughput while maintaining accuracy. Over the last two decades, automatic cell counters have undergone significant improvements due to introduction of new technologies. Coulter technology using impedance for counting particles is one of the most popular automated methods. With the help of modern micro-fabrication techniques, it is now possible to fabricate sub-micrometer apertures for counting and detecting particles as small as DNA molecules. Two decades after the advent of Coulter technology, flow cytometry developed as a result of advancements in the fields of optics, antibody production, and fluorochrome discovery. The benefits of flow cytometers over coulter counters are in terms of their ability to distinguish cells based on multiple parameters such as a wide array of cellular markers. This technology was quickly adopted commercially. Another commonly used technology is referred to as image-based cytometry. With an increase in computing capabilities and high-quality camera sensors, there has been an increase in image based analysis for cell counting. This technology is implemented through high-resolution imaging or microscopy of single cells in static and flowing environments. Using this approach, image based morphological data from a heterogeneous cell suspension is obtained using powerful segmentation and feature extraction algorithms.

Microfluidic cytometers have brought accurate and high throughput way to enumerate and characterize cells on devices with a small footprint. Advancements in microfabrication methods have allowed integration of electrodes, optics, and various detection systems on a chip. Miniaturization will eventually diminish the need for benchtop cytometry by replacing these relatively large devices with portable or even wearable platforms that provide qualitative information about the various cell types within a heterogeneous sample such as blood. In addition, such platforms would also enumerate rare cells, such as CTCs and other cell irregularities within biological fluids. Providing diagnostic information from portable home based blood counting devices without the need for a centralized diagnostic laboratory will provide user value and generate commercial interest for medical device manufacturers. These miniaturized cell counters could be accessible at low cost like modern blood sugar monitors (i.e., Blood Glucose Meter) and act as an indicator for disease status at point-of-care settings.

## Author Contributions

AV, AM, and MQ: designed the research. AV and AM: wrote the manuscript. MQ: supervised the work and finalized the manuscript.

### Conflict of Interest Statement

The authors declare that the research was conducted in the absence of any commercial or financial relationships that could be construed as a potential conflict of interest.
